# Anthropogenic and Climatic Variables Jointly Shape the Global Geographical Pattern of Hybrid Plant Diversity

**DOI:** 10.1002/ece3.71512

**Published:** 2025-05-29

**Authors:** Sirui Song, Yadong Zhou

**Affiliations:** ^1^ School of Life Sciences Nanchang University Nanchang China

**Keywords:** anthropogenic variables, climate, diversity, geographical pattern, hybrid plants, parental species

## Abstract

The emergence and dispersal of hybrid plants are influenced by a complex interplay of climatic, environmental, and anthropogenic factors. However, systematic investigations into the global patterns of hybrid plant diversity and their underlying drivers remain scarce. In this study, we compiled a comprehensive dataset encompassing 3543 hybrid plants and their parental species across 274 geographical regions. We analyzed the species richness, species density (SD), and hybridization index (which quantifies the spatial overlap between hybrids and their parental species), along with their associations with climatic, vegetation, and anthropogenic variables. Our results reveal that hybrid plant diversity is highest in Europe and Japan, whereas Africa, Oceania, and the Atlantic Ocean exhibit significantly lower diversity. Notably, hybrid plant diversity shows strong correlations with both anthropogenic and climatic factors, with anthropogenic influences playing a more dominant role in shaping global hybrid distributions. This is particularly evident in hybrid‐rich regions such as Europe and Japan, where locally distributed hybrids display reduced overlap with their parental species—a pattern likely driven by human‐mediated dispersal or other anthropogenic activities. Our findings provide novel insights into the global diversity and dispersal dynamics of hybrid plants.

## Graphical abstract

The highest levels of hybrid richness and density were observed in Europe and Japan, whereas the lowest levels were recorded in Africa, Oceania, and the Atlantic Ocean. Furthermore, anthropogenic and environmental variables exert a more pronounced influence on the global distribution pattern of hybrids, with anthropogenic variables being particularly significant. Based on these findings, and considering the recent history of human‐mediated species introductions, we predict that the abundance and density of hybrids will increase in the coming centuries, especially in countries with well‐developed trade, transport, and economic growth.

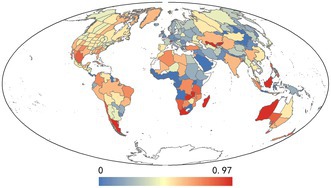

## 1 Introduction

A comprehensive understanding of global plant diversity (PD) and its effective utilization requires an in‐depth examination of the geographical distribution of plant taxa. Extensive research has been conducted on various plant groups at regional and global scales, including angiosperms (Moulatlet et al. 2023; Qian and Qian 2023), seed plants (Qian et al. 2019), vascular plants (Möls et al. 2013; Kreft and Jetz 2007), and bryophytes (Carter et al. 2022; Mateo et al. 2016; Möls et al. 2013). Hybrid plants, which arise from interspecific hybridization, are influenced by a combination of climatic, environmental, and anthropogenic factors (Campbell and Wendlandt 2013; Taylor et al. 2015; Staude and Ebersbach 2023). Nevertheless, while there are numerous studies that have explored the geography of hybrid plants, these have generally focused on a specific case for a particular hybrid or hybrids, rather than a broader synthesis of global patterns (Jacquemyn et al. 2012; Sujii et al. 2019).

Interspecific hybridization occurs when genetically distinct taxa interbreed, producing viable offspring with both genetic divergence and phenotypic differentiation (Mallet 2005). This process can accelerate speciation by generating novel allelic combinations, thereby enhancing genetic diversity (Abbott et al. 2013; Anderson and Stebbins 1954; Marques et al. 2019; Thomas 2015). The role of hybridization in plant evolution is increasingly recognized (Mallet 2005; Whitney et al. 2010), especially by rearranging inherited genetic variation, which allows natural selection to form new species (Grant and Grant 1992; Seehausen 2004). Vital processes including speciation, adaptive radiation, and evolution and diversification have all been connected to hybridization (Soltis and Soltis 2009; Abbott et al. 2013; Yakimowski and Rieseberg 2014; Marques et al. 2019).

Climate change, industrialization, habitat degradation, and global trade and travel have facilitated the worldwide dispersal of plants, breaking down historical geographical and ecological barriers that once restricted gene flow between closely related species (Mooney and Cleland 2001). As these barriers diminish, opportunities for hybridization have expanded dramatically (Mable 2013; Chunco 2014; Brennan et al. 2014; Taylor et al. 2015). A major driver of this phenomenon is human‐mediated species transport—whether intentional or accidental—which has occurred for millennia but has intensified rapidly in regions such as North America and Europe (Hulme et al. 2008). In today's globalized world, the spread of non‐native species is expected to increase further due to expanding international trade and travel (Hulme et al. 2008). Human‐assisted dispersal operates through multiple pathways, including deliberate introductions (e.g., horticultural trade), facilitated dispersal (e.g., along roadsides and railways), and accidental long‐distance dispersal events that translocate terrestrial plant species across oceanic barriers (Vallejo‐Marín and Hiscock 2016). Although long‐distance dispersal events are rare, theoretical models suggest they can significantly enhance plant migration rates (Higgins and Richardson 1999).

In addition to anthropogenic variables, climate variables also play a significant role in hybrid plant formation, though their influence is generally weaker than that of anthropogenic drivers. Climate change induced by global warming may expand sympatric zones between species or bring previously isolated taxa into contact by altering their geographic ranges (Hoffmann and Sgrò 2011; Brennan et al. 2014). For instance, variations in temperature or precipitation can lead to a greater overlap of ranges and the development or growth of hybrid zones (Campbell and Wendlandt 2013; Taylor et al. 2015). Since hybrid zones often form at the boundaries of parental species' distributions, changes in these geographic limits can significantly affect the location and extent of hybrid regions (Chunco 2014). Furthermore, climate change may promote the spread of invasive populations, potentially leading to hybridization events between native species and invasive taxa (Vallejo‐Marín and Hiscock 2016).

To harness hybrid distribution mechanisms for human benefit, it is essential to investigate the global geographic patterns of hybrid PD and their underlying anthropogenic and climatic drivers. Although recent studies have explored various aspects of hybridization—including its role in speciation (Abbott et al. 2013) and evolutionary processes (Anderson and Stebbins 1954)—a systematic analysis of hybrid plant distribution patterns and their key determinants (e.g., anthropogenic and climatic variables) remains lacking. In this study, we examine the global distribution of hybrid plants and assess the influence of local species diversity, anthropogenic factors, and climate. We specifically address the issue of what variables most affect the patterns of hybrid distribution.

## 2 Materials and Methods

### 2.1 The Dataset of Hybrid Plants

Our study analyzed 274 geographical regions based on the World Geodetic System 1984 (WGS84) Coordinate System. However, not all regions within the WGS84 framework were included in the analysis. Some were excluded due to the absence of hybrid plant occurrences, whereas others were omitted because of missing climatic, anthropogenic, or local PD data. Species lists of hybrid plants in these regions were compiled on Plants of the World Online (http://www.plantsoftheworldonline.org), the World Checklist of Selected Plants (WCSP), and World Plants (https://www.worldplants.de).

**FIGURE 1:**
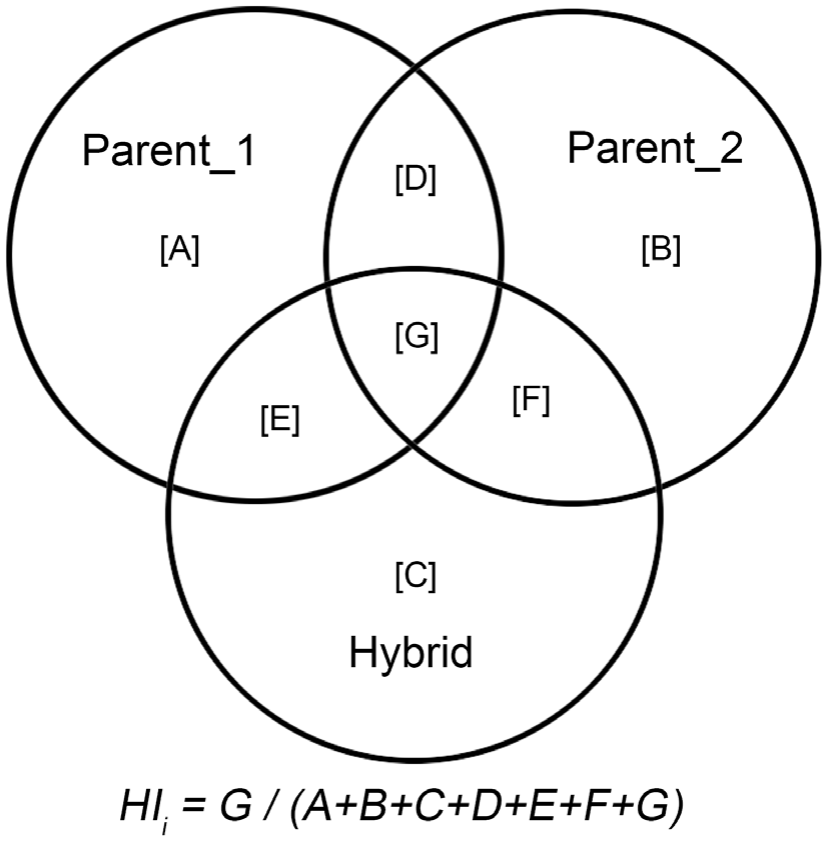
Graphical representation of the calculation of hybridization index (HI) in this study, which represents the degree of overlap between hybrid plants and their two parents in the natural distribution area.

From Plants of the World Online, we obtained a database of 3543 hybrid plants and their respective parental species. We identified the natural distribution ranges of each parental species (excluding artificially introduced ranges) and determined the overlapping areas between hybrids and their parents. The hybridization index (HI) was calculated as the ratio of the overlapping area to the total distribution area of the hybrid and its parents (Figure 1; Table S1). This index quantifies the degree of range overlap between hybrids and their parental species.

### 2.2 Climatic and Anthropogenic Variables

We evaluated the influence of local PD, anthropogenic factors, and climate on the global distribution of hybrid plants (Table S1). Anthropogenic factor data came from the human development index (HDI) (https://ourworldindata.org/) and the human footprint (HF) (https://sedac.ciesin.columbia.edu/). Data sources for local PD included the normalized difference vegetation index (NDVI) (http://poles.tpdc.ac.cn/zh‐hans/) and PD (https://gift.uni‐goettingen.de/shiny/predictions/). The 19 bioclimatic variables data at the resolution of 30 arc‐seconds were obtained from the WorldClim database (http://www.worldclim.org, version 2.1) (Fick and Hijmans 2017). Concurrently, principal component analyses were performed on these 19 climate variables to eliminate highly correlated variables. The principal component analysis of the 19 climate variables demonstrates that PC1 and PC2 account for 77.76% of the variability in the data (Table S2).

### 2.3 Data Analysis

We conducted a statistical analysis of 3543 hybrid plants across 274 regions to assess species richness (SR), species density (SD), and the HI, which quantifies the degree of overlap between hybrids and their parental species (Table S1). The global distribution of hybrid plants in terms of SD, SR, and HI was then mapped using ArcGIS software. Concurrently, the values of HI were ordered from lowest to highest, and the regions comprising the top and bottom 50% of HI values were identified. Subsequently, the global distribution patterns of SD and SR in the regions comprising the top 50% of HI values and the global distribution patterns of SD and SR in the regions comprising the bottom 50% of HI values were plotted. Additionally, the variation of SD, SR, and HI with latitude and longitude was plotted to facilitate a more detailed observation of the global distribution patterns of hybrid plants. In order to gain a more comprehensive understanding of the influence of climatic variables, plant and vegetation, and anthropogenic variables on hybrid distribution patterns, and to ascertain the relative impact of these three variables, we conducted an explanatory variance decomposition of the three variables. Subsequently, the impact of the aforementioned three variables on the richness and density of hybrid plants in each region, as well as their influence on HI, was examined. Subsequently, the synchronous autoregressive (SAR) error models were employed to calculate the normalized regression coefficients and determination coefficients (*R* ^2^) for SR, SD, and HI based on climate (PC1 and PC2), PD, NDVI, HF, and HDI.

## 3 Result

### 3.1 Global Pattern of Hybrid Plants

**FIGURE 2:**
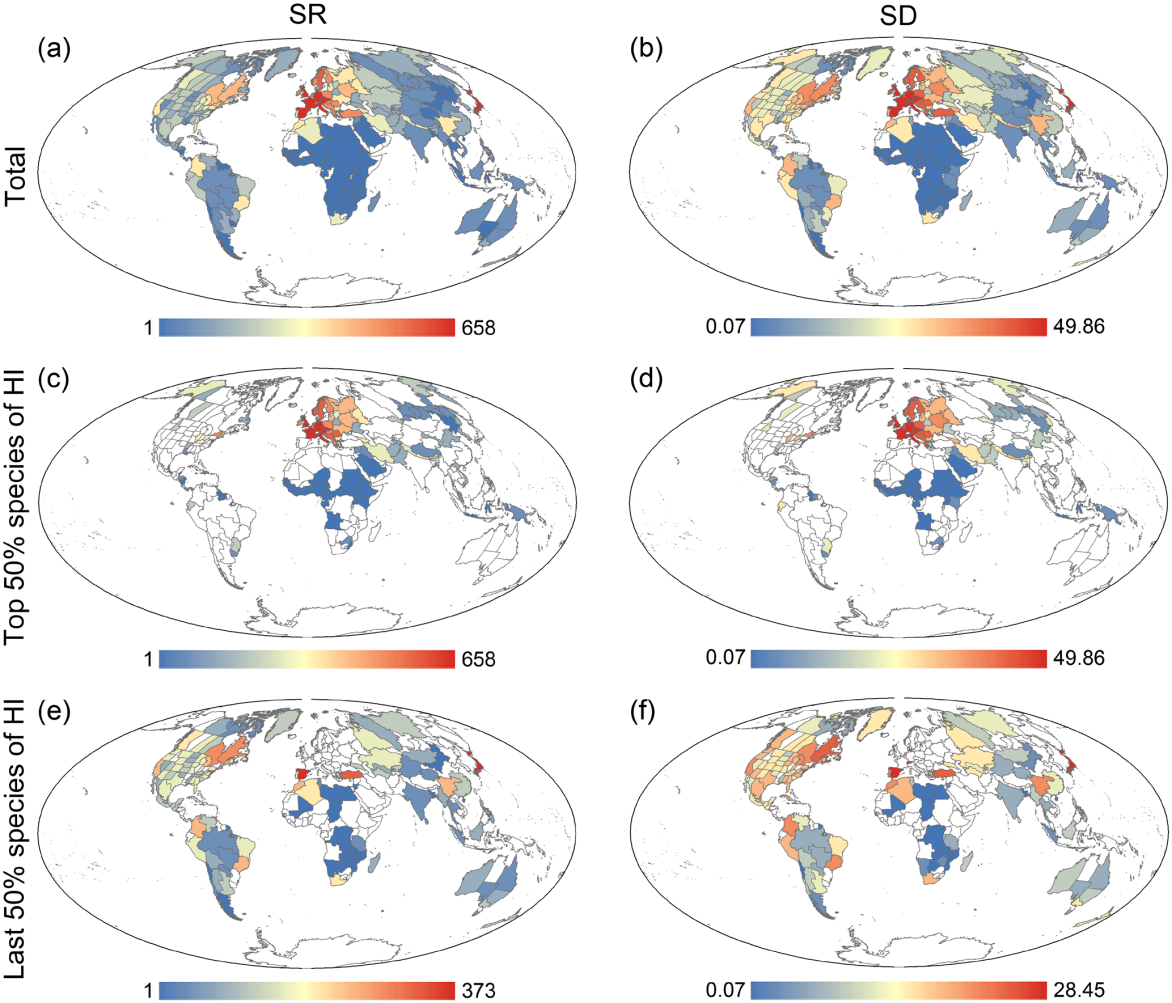
Geographical patterns of species richness (SR) and species density (SD) of hybrid plants. (a) and (b) represent all these hybrid plants; (c) and (d) represent the top 50% of hybrid plants of the hybridization index (HI); (e) and (f) represent bottom 50% of hybrid plants of the hybridization index (HI).

**FIGURE 3:**
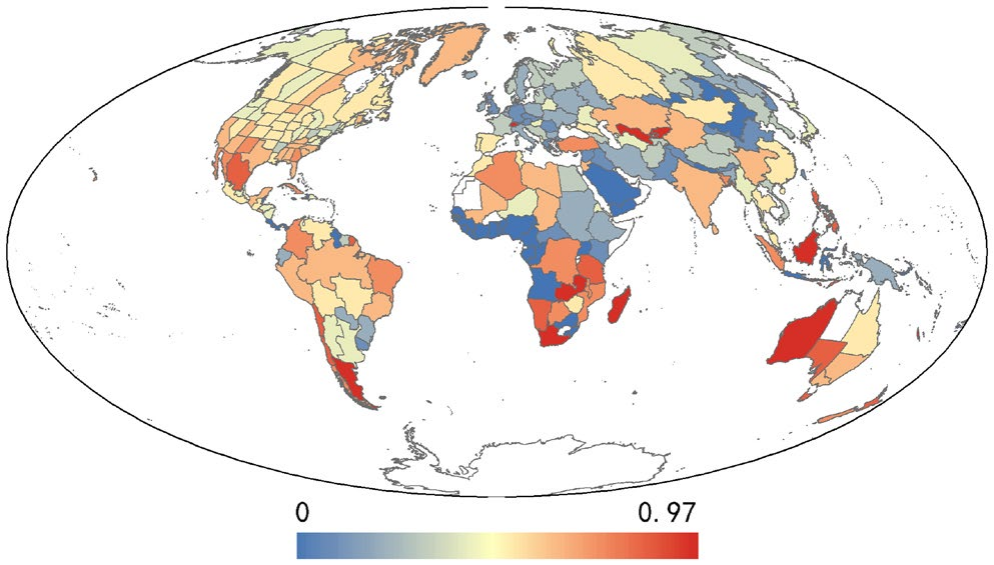
Geographical pattern in mean hybridization index (HI) in this study.

The global distribution of hybrid plants revealed that SR and SD followed similar trends. High values of SD and SR were concentrated primarily in Europe and Japan, whereas low values predominated in Africa, Oceania, and the central Eurasian continent (Figure 2a,b). In contrast, the HI exhibited an inverse trend, with higher values predominantly in Southern Africa and Oceania and lower values in Europe and Japan (Figure 3). To further analyze spatial patterns, regions were ranked by HI in ascending order, and the top and bottom 50% were examined for SD and SR trends. The results showed that the SR and SD trends remained almost identical in both the first and second 50% of the area. The initial 50% of the regions revealed that Europe exhibited the highest SD and SR, indicating that hybrid plants are predominantly distributed within this region (Figure 2c,d). Conversely, the subsequent 50% of regions demonstrated that Japan and North America exhibited the highest SD and SR, suggesting that hybrid plants are primarily distributed in these regions (Figure 2e,f).

**FIGURE 4:**
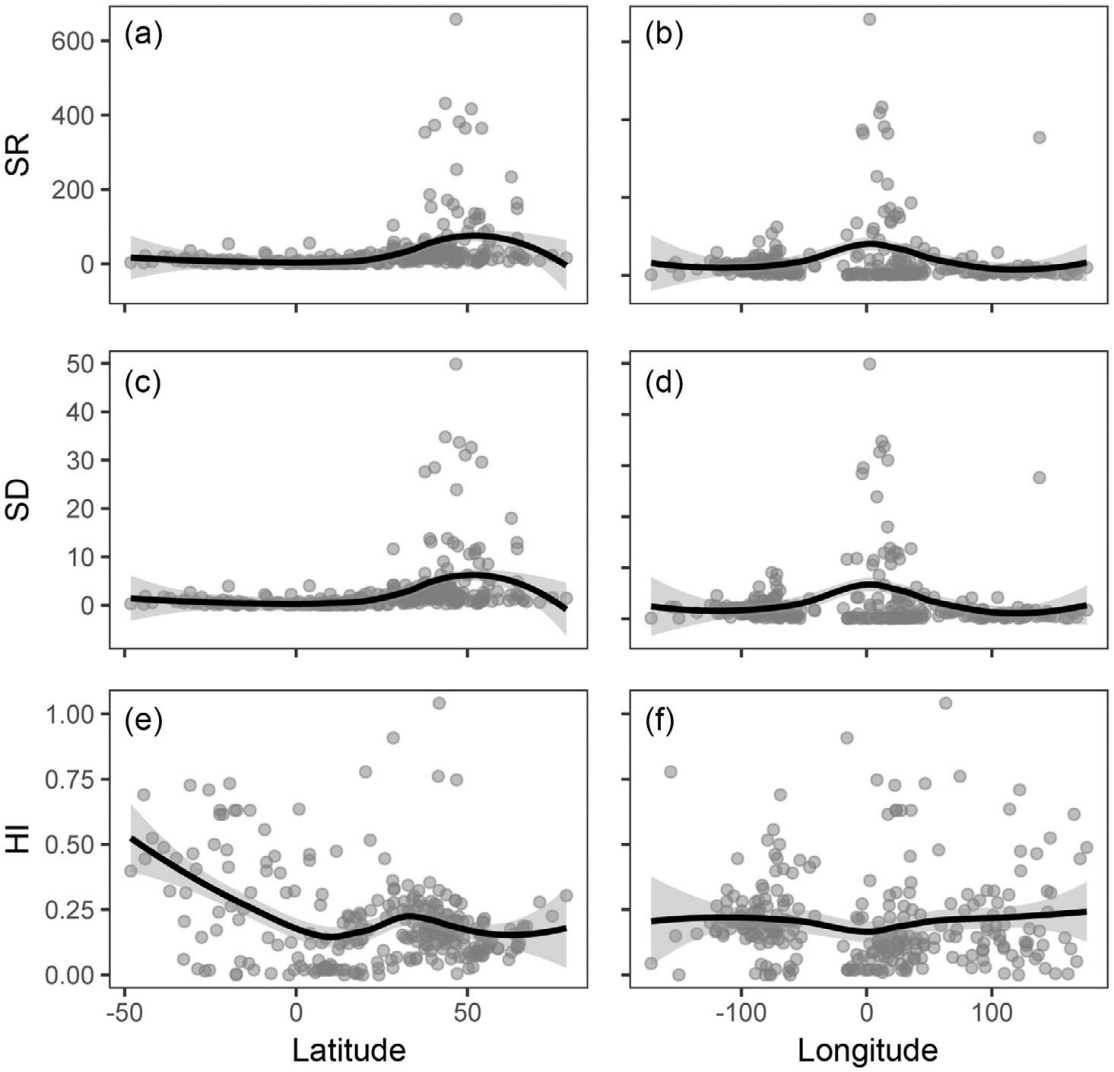
Line plots of species richness (SR), species density (SD), and hybrid‐parent overlap (HI) with latitude and longitude.

By using latitude and longitude to do linear regression analysis with the values of SD, SR, and HI, it can be understood that SD and SR still have the same trend, and they both get the maximum value at about 40°–60° N, −20° E to 20° W, and remain stable and low at the rest of the latitude and longitude (Figure 4a–d). The trend of HI continues to be concavely opposite to that of SD and SR, and the value of HI gradually rises to the maximum in 0°–50° S, and remains basically constant in 0°–50° N except for a slight peak at about 40° N; the value of HI remains essentially constant in longitude, except for a slight minimum at about 0° (Figure 4e,f).

### 3.2 Variables Affecting the Global Distribution Pattern of Hybrid Plants

The normalized regression coefficient and determination coefficient (*R* ^2^) of the SAR error model were calculated based on climate variables (PC1 and PC2), PD, NDVI, HF, and HDI (Table 1). It is evident that anthropogenic variables exert the most significant influence on the variation in SD, SR, and HI. Specifically, among these variables, the HDI has the greatest impact on the variation in SD, SR, and HI; meanwhile, environmental variables also play a substantial role in driving this variation. In comparison, plant and vegetation have the least influence on SD, SR, and HI, and the influence of PD on SD, SR, and HI is much greater than that of NDVI (Table 1).

**TABLE 1:** Standardized regression coefficients and coefficients of determination (*R* ^2^) from simultaneous autoregressive (SAR) error models for SR, SD, and HI, with the climate (PC1 and PC2), plant diversity (PD), normalized difference vegetation index (NDVI), human footprint (HF), and human development index (HDI).

	SR	SD	HI
Climatic variables			
PC1	−0.152^ns^	−0.146^ns^	0.019^ns^
PC2	−0.100^ns^	−0.104^ns^	0.253***
Plant and vegetation			
PD	0.122^ns^	0.121^ns^	0.082^ns^
NDVI	−0.009^ns^	−0.008^ns^	−0.023^ns^
Anthropogenic variables			
HF	0.128^ns^	0.148*	−0.071^ns^
HDI	0.197*	0.209*	0.347***
*R* ^2^	0.242	0.274	0.195

*p* value: *** < 0.001, * < 0.05, 0.05 < ns < 1.

**FIGURE 5:**
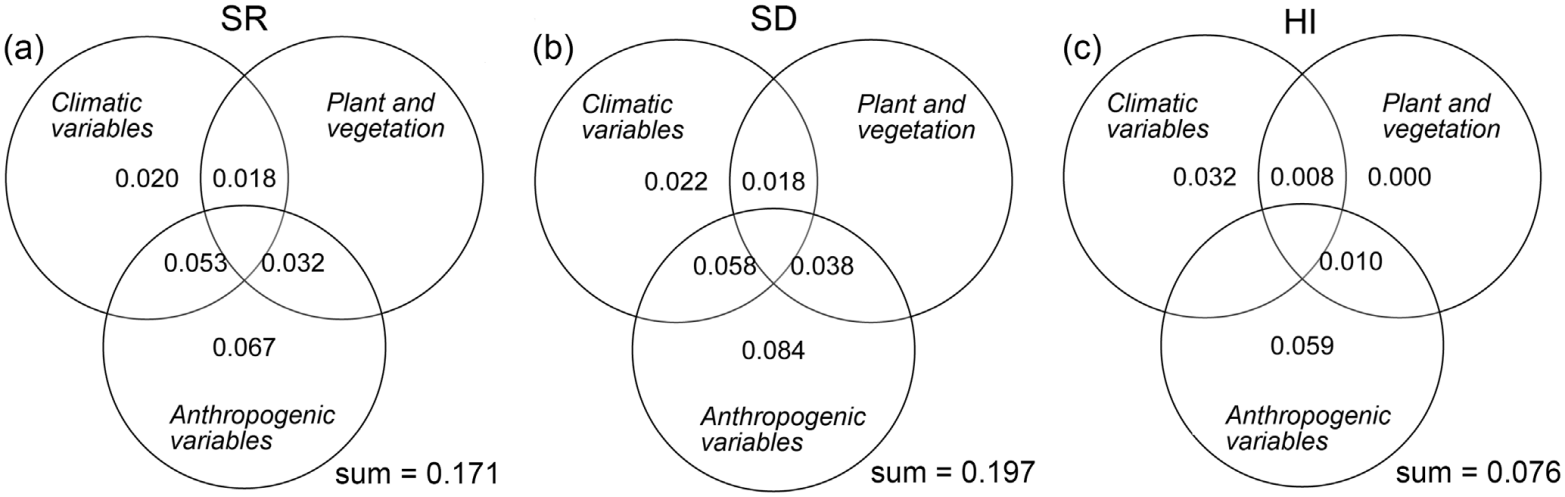
Pure and shared effects of three sets of explanatory variables on indicators of global regional hybrid plant systems. The detailed information of the three explanatory variables is shown in Table 1.

Variables affecting the global distribution pattern of hybrid plants are analyzed in three ways: anthropogenic variables, climatic variables, and plant and vegetation. When all three sets of explanatory variables were considered, it was found that the aforementioned three variables were responsible for explaining 17.1% of the SR variation, 19.7% of the SD variation, and only 7.8% of the HI variation (Figure 5). A total of 17.1% variation of SR was explained by variables, with anthropogenic variables accounting for less than half and climatic variables accounting for a relatively minor proportion. It is unlikely that plant and vegetation is the sole factor responsible for the observed variation of SR (Figure 5a). The combined influence of climate and anthropogenic variables on SR change is estimated to account for 5.3% of the total observed variation (Figure 5a). The combined effect of plant and vegetation and anthropogenic variables accounted for 3.2% variation of SR, whereas the joint influence of climatic variables and plant and vegetation explained 1.8% variation of SR (Figure 5a). An analysis of the data revealed that 19.7% variation of SD were explained by variables, of which anthropogenic variables accounted for nearly half, and climatic variables accounted for a small part. It was also found that plant and vegetation could not explain the variation of SD alone (Figure 5b). Climate and anthropogenic variables jointly explained 5.8% variation of SD, plant and vegetation and anthropogenic variables jointly explained 3.8% variation of SD, and climate variables and plant and vegetation jointly explained 1.8% variation of SD (Figure 5b). Approximately 7.8% of the change in HI is explained by variables, with approximately 80% of this explained by anthropogenic variables independently and 3.2% explained by climate variables alone (Figure 5c). The remaining 1.0% of the change in HI is explained by the joint effect of anthropogenic variables and plant and vegetation, whereas the remaining 0.8% is explained by the joint effect of plant and vegetation and climate variables (Figure 5c).

## 4 Discussion

Although hybrid plants constitute a relatively small proportion of the global flora, they play a crucial role in evolution and diversification (Anderson and Stebbins 1954; Seehausen 2004), adaptive radiation (Yakimowski and Rieseberg 2014; Marques et al. 2019), species formation (Soltis and Soltis 2009; Abbott et al. 2013), and ornamental horticulture (Allard 1999). Previous research has concentrated on the influence of hybrid plants on species formation (Abbott et al. 2013), the facilitative impact of hybridization on evolutionary processes (Anderson and Stebbins 1954), and the correlates of hybrid plants (Mitchell et al. 2019). In contrast, this study employs large‐scale data analysis to examine the global distribution patterns of hybrid PD and their parental species. Our findings reveal distinct geographical trends in hybrid PD and quantify the relative contributions of anthropogenic and climatic factors to hybrid formation.

### 4.1 Global Patterns and Driving Mechanisms of Hybrid Plant Richness and Density

Our results indicate that hybrid SR and SD are highest in Europe and Japan, whereas the lowest levels are found in Africa, Oceania, and the Atlantic Ocean. Several factors may explain this pattern: first, Europe and Japan are economically developed, easily accessible, and have a high level of human activities. Second, the climate in these regions is favorable for hybrid growth, that is, Europe and Japan have temperate continental and subtropical monsoon climates, respectively (Köppen 1936), which create optimal environments for plant growth, thereby supporting elevated diversity levels. The climates of Europe and Japan are characterized by a high index of seasonal temperature variability, with distinct seasons and pronounced thermal fluctuations throughout the year. This habitat complexity favors the development of more biologically diverse communities, in part because of the higher abundance and diversity of ecological niches in heterogeneous environments (MacArthur and MacArthur 1961) and because the temporal stability of ecosystems (e.g., temporal variability, resistance, and resilience) increases with SR and age of the community (Isbell et al. 2015; Schnabel et al. 2021; Tilman et al. 2006; Van Moorsel et al. 2021; Wagg et al. 2017, 2022). This in turn favors local ecosystem stability and hybrid plant growth. The indices for PD and homogeneity are higher in Europe and Japan. Furthermore, an increase in PD has been demonstrated to enhance the functionality of individual ecosystems, particularly with regard to plant productivity (Köppen 1936). In addition, PD exhibits multiple functions, a phenomenon known as ecosystem multifunctionality, which is particularly conducive to hybrid growth (Fanin et al. 2018; Hautier et al. 2018; Schuldt et al. 2018). The underlying reason for this is that species‐rich, old, mature plant communities benefit more from complementary effects and favorable plant–soil interactions (Cardinale et al. 2007; Reich et al. 2012; Thakur et al. 2021), which makes them more resistant to disturbance (Bennett et al. 2020; Craven et al. 2016; Wang et al. 2021) and therefore more stable over time (Ren et al. 2022; Wagg et al. 2022; Yang et al. 2018). At last, it is evident that plant species are adapting their distributions in response to ongoing climatic changes at the regional level (Parmesan and Yohe 2003; Root et al. 2005; Walther et al. 2005; Lavergne et al. 2006). During the Last Glacial Maximum (LGM, approximately 21,000 years ago), the majority of European plant species were confined to latitudes south and east of the Scandinavian ice cap. However, following the LGM, a period of warming permitted the expansion of species northward (Normand et al. 2011). This migration causes plants from different regions to meet and form hybrid plants. Concurrently, hybrid plants typically exhibit hybrid heterosis, which denotes the phenomenon wherein a heterozygous hybrid displays enhanced performance relative to its parental species (Liu et al. 2020). This phenomenon is highly advantageous for plant reproduction and adaptation to environmental alterations (Liu et al. 2020), thereby enabling the plant to more effectively adapt to novel environments and effectively cope with harsh climatic conditions. As a result, in challenging environments, plants are more likely to engage in hybridization. Given that hybridization is a driver of species range expansion (Pfennig et al. 2016), the progeny of these hybrid plants tend to exhibit greater distributional and reproductive breadth than their parent species.

Furthermore, we hypothesize that global hybrid distribution patterns may be closely linked to regional disparities in scientific capacity, particularly uneven progress in plant taxonomy. Europe and Japan serve as prime examples: Europe is the birthplace of modern plant taxonomy, with foundational work dating back centuries (Tutin et al. 1980). At the same time, Japan's botanical research advanced significantly during the Meiji Restoration, driven by scientific modernization (Barnes 2001). This suggests that Europe and Japan may have an advantage in the field of plant taxonomic research, an advantage that allows local scholars to carry out more fine‐grained species delimitation of plant taxa. Whereas elsewhere certain morphologically variable groups of plants may be grouped into a single taxonomic unit, European and Japanese researchers, with their more advanced taxonomic methods and high intensity of scientific investment, are often able to identify separate species. This discrepancy in taxonomic perception is directly reflected in the hybrid data, resulting in these two regions showing significantly higher levels of hybrid formation rates and hybrid PD than other regions.

### 4.2 Global Pattern of Hybridization Index

The value of HI represents the magnitude of overlap between the hybrid distribution site and the parental distribution site. In comparison to previous studies on the distribution patterns of different plant taxa, which were frequently conducted by analyzing SR (Chen et al. 2021, 2023; Dainese et al. 2013; Zhang et al. 2022) and PD (Zhang et al. 2022), HI is a more pertinent approach as it considers not only the hybrid plants themselves but also their parents. The impact of natural hybridization on our understanding of taxonomic relationships (reticulate versus divergent evolution) is significant (Viljoen et al. 2006). However, much of the current research focuses on the analysis of hybrid phenotypes and the estimation of the degree of divergence between hybrid taxa (Rieseberg et al. 2000), rather than on the identification of whether the hybrid is of natural or anthropogenic origin. The HI provides a tool for estimating whether a hybrid is of natural or anthropogenic origin and for determining the proportion of natural hybrid plants among hybrid plants in a region. The magnitude of the HI value for a single species is indicative of the probability that the species originated from natural hybridization. Consequently, the larger the HI value for a single species, the greater the overlap between the hybrid's range and that of its parents, and the greater the likelihood that it originated from natural hybridization. The converse is true for hybrid plants that are more likely to be associated with anthropogenic activities. Conversely, the magnitude of the mean HI value within a given region is indicative of the proportion of natural hybrid plants present. At the same time, a larger mean HI value within a region is indicative of a higher proportion of natural hybrid plants. Conversely, the proportion of natural hybrid plants is inversely proportional to the proportion of anthropogenic hybrid plants in the region. The trend of HI is almost the inverse of that of SR and SD, with larger values observed in southern Africa, Oceania, and the southern Atlantic Ocean, and the lowest values in Europe and Japan. The observed trends in HI values suggest that hybrid plants distributed in Europe and Japan have a relatively limited range of overlap with their parental species. However, the highest levels of hybrid richness and hybrid density were observed in these regions. It can be postulated that the ability of the parental species to crossbreed across geographical barriers and disperse their progeny over vast distances is a consequence of human activities.

The application of three sets of explanatory variables, namely anthropogenic variables, climatic variables, and plant and vegetation, to elucidate the variations in SD, SR, and HI revealed that anthropogenic variables exerted a more pronounced influence on the global distribution pattern of hybrid plants. This manifested as hybrid PD and density being higher in regions characterized by elevated HDI, dense population, and well‐developed transport infrastructure, such as Europe and Japan. In accordance with prior research indicating that hybrid plants thrive in disturbed ecosystems (Anderson 1948; Wiegand 1935), Staude also found that neophyte‐native hybrid plants in Britain typically emerged in areas with a more pronounced human impact than their parental species (Staude and Ebersbach 2023).

The Hybrid Index (HI) was sorted in ascending order, and regions with HI values in the upper and lower 50% were selected for comparative analysis. The results demonstrate that in regions with the highest 50% of HI values, hybrid plants are predominantly distributed across Europe. This may be attributed to the fact that the fifteenth to seventeenth centuries marked the advent of the Age of the Great Voyages, during which European maritime nations undertook expeditions to explore the globe in search of lucrative trade commodities, including gold and spices (Royle 2009). The subsequent globalization of trade became a hallmark of the era (Domínguez‐Delmás et al. 2015). For instance, Christopher Columbus's 1492 voyage catalyzed unprecedented intercontinental exchanges between the Old and New Worlds (Nunn and Qian 2010). The Great Columbian Trade also facilitated the dissemination of crops across the Atlantic, including potatoes, sweet potatoes, maize, and cassava, as well as the introduction of tomatoes, peppers, and cocoa into Old World countries such as Europe (Nunn and Qian 2010). These introductions significantly increased the likelihood of interspecific hybridization between native European flora and exotic species. This was succeeded by the Industrial Revolution in Europe, which commenced in Britain between the 1760s and 1780s (Buckberry and Crane‐Kramer 2022). The revolution precipitated sweeping technological, environmental, and societal changes (Buckberry and Crane‐Kramer 2022), further accelerating Europe's economic growth. However, with the growth of global exploration and trade in the 1800s and the increased prosperity and leisure time generated by industrialization (Dozier 1999), horticulture in Europe also developed rapidly. A considerable number of currently invasive species were deliberately introduced as exotic pets or ornamentals (Cassey et al. 2004; Semmens et al. 2004; Duggan et al. 2006). Notably, the horticultural trade has been a primary vector for the deliberate translocation of species beyond their native ranges (Mack 2003). In fact, most alien plant species in various regions trace their origins to horticultural introductions (Groves 1998; Mack and Erneberg 2002; Pyšek et al. 2002), thereby substantially elevating the probability of hybrid formation. The remaining 50% of hybrid cases are predominantly found in Japan and North America. This distribution pattern can be linked to historical developments such as the Meiji Restoration (1860s), during which the Japanese government implemented sweeping industrial, military, and constitutional reforms to modernize the nation (Murata 2001), triggering rapid socioeconomic growth. Similarly, the United States—the primary focus of North America in this context—was established in 1776 but lagged behind Europe in horticultural development by approximately a century (Manks 1968). It can be observed that the development of Japan and North America is more recent than that of Europe, and the emergence of hybrid plants in these regions may be predominantly influenced by climatic variables. Nevertheless, while environmental variables play a significant role in shaping hybrid plant distributions globally, their impact remains secondary to anthropogenic drivers.

### 4.3 Climate and Human Disturbance Jointly Affect Hybrid Plant Diversity

Concurrently, the interplay between anthropogenic and environmental variables has resulted in a substantial impact on the global distribution of hybrid plants. Lawrence and Fraser (2020) observed that SR peaks at equatorial latitudes, yet hybrid diversity follows a distinct pattern, with notable concentrations in Europe and Japan. This demonstrates that regions with high PD do not necessarily yield a greater number of hybrid plants. The formation of hybrid plants should not be considered in isolation, but rather in conjunction with other variables, including climatic and anthropogenic influences, as well as the combined effects of climatic and anthropogenic variables.

Both climate change and human disturbance can promote hybridization through distinct mechanisms. Among these, human activities mediate hybridization through physical alteration of habitats (Kimura and Munehara 2010) or intentional and unintentional introduction of alien species (Rhymer and Simberloff 1996; Walters et al. 2008). Climate change facilitates hybridization by breaking down physical, temporal, and behavioral barriers to reproduction between species (Chunco 2014). Critically, these factors do not operate independently—climate change can amplify human‐mediated hybridization. For example, *Banksia hookeriana* and *Banksia prionotes*, two Australian dune plants with shared pollinators, naturally exhibit asynchronous flowering, preventing hybridization (Lamont et al. 2003). However, human disturbances (e.g., roadside and railway construction) disrupt soil conditions, prolonging *B. prionotes* flowering and accelerating *B. hookeriana* blooming, thereby enabling crossbreeding (Lamont et al. 2003). Concurrently, climate warming can change the phenology of plants, which can also lead to earlier and longer flowering times (Crimmins et al. 2010). Thus, the synergistic effects of global climate change and anthropogenic perturbations may enhance human‐mediated hybridization.

## 5 Conclusion

This study investigated the global distribution patterns of hybrid plants and their underlying drivers from a macroecological perspective, providing insights into their potential ecological consequences. Our findings reveal that hybrid richness and density are highest in Europe and Japan, whereas Africa, Oceania, and the Atlantic Ocean exhibit the lowest levels. Both anthropogenic and environmental factors strongly influence these patterns, with human activities emerging as the dominant driver. Given historical trends in human‐mediated species introductions, we project a substantial increase in hybrid plant abundance and density in the coming centuries, particularly in regions with well‐developed trade networks, transportation infrastructure, and economic growth. However, these results primarily demonstrate broad‐scale correlations rather than mechanistic relationships, highlighting the need for further research to elucidate the precise pathways through which anthropogenic and environmental factors shape hybrid distributions. Several methodological limitations must be acknowledged. First, the use of WGS84 grid cells—rather than ecologically meaningful boundaries—may lead to range misestimations by incorporating unsuitable habitats. Second, data collection was limited to three major databases (POWO, WCSP, and World Plants), which may introduce taxonomic biases in hybrid identification and parentage assignment. These constraints suggest that the study serves better as a preliminary assessment of macro‐scale hybrid patterns than as a precise predictive model. Future research should integrate high‐resolution habitat modeling with molecular validation to enhance accuracy. Despite these limitations, the findings provide a foundation for informed conservation strategies. In plant trade, hybrids may be commercially valuable but could also introduce invasive risks, necessitating stricter quarantine measures in high‐risk zones. For habitat restoration, certain hybrids may aid ecosystem recovery (e.g., as pioneer species), though their genetic integrity and ecological impacts require careful evaluation. In invasive species management, understanding hybrid distributions can improve early detection and control, given their enhanced adaptability. Thus, while this study advances broad‐scale understanding, targeted follow‐up work is essential to translate patterns into actionable policies.

## Author Contributions

**Sirui Song:** data curation (lead), formal analysis (equal), investigation (equal), methodology (equal), resources (equal), writing – original draft (equal). **Yadong Zhou:** formal analysis (equal), funding acquisition (lead), methodology (equal), project administration (lead), writing – review and editing (lead).

## Conflicts of Interest

The authors declare no conflicts of interest.

## Supporting information


Table S1

Table S2

## Data Availability

The data supporting the findings of this study are included within the main document and Supporting Information.

## References

[ece371512-bib-0001] Abbott, R. , D. Albach , S. Ansell , et al. 2013. “Hybridization and Speciation.” Journal of Evolutionary Biology 26: 229–246. 10.1111/j.1420-9101.2012.02599.x. 23323997

[ece371512-bib-0002] Allard, R. W. 1999. Principles of Plant Breeding. 2nd ed. Wiley.

[ece371512-bib-0003] Anderson, E. 1948. “Hybridization of the Habitat.” Evolution 2, no. 1: 1–9. 10.1111/j.1558-5646.1948.tb02726.x.

[ece371512-bib-0004] Anderson, E. , and G. L. Stebbins . 1954. “Hybridization as an Evolutionary Stimulus.” Evolution 8: 378–388. 10.1111/j.1558-5646.1954.tb01504.x.

[ece371512-bib-0006] Barnes, P. 2001. “Japan's Botanical Sunrise: Plant Exploration Around the Meiji Restoration.” Curtis's Botanical Magazine 18: 117–131.

[ece371512-bib-0007] Bennett, J. A. , A. M. Koch , J. Forsythe , N. C. Johnson , D. Tilman , and J. Klironomos . 2020. “Resistance of Soil Biota and Plant Growth to Disturbance Increases With Plant Diversity.” Ecology Letters 23: 119–128. 10.1111/ele.13408. 31650676

[ece371512-bib-0008] Brennan, A. C. , G. Woodward , O. Seehausen , et al. 2014. “Hybridization due to Changing Species Distributions: Adding Problems or Solutions to Conservation of Biodiversity During Global Change?” Evolutionary Ecology Research 16: 475–491.

[ece371512-bib-0009] Buckberry, J. , and G. Crane‐Kramer . 2022. “The Dark Satanic Mills: Evaluating Patterns of Health in England During the Industrial Revolution.” International Journal of Paleopathology 39: 93–108. 10.1016/j.ijpp.2022.10.002. 36335796

[ece371512-bib-0010] Campbell, D. R. , and C. Wendlandt . 2013. “Altered Precipitation Affects Plant Hybrids Differently Than Their Parental Species.” American Journal of Botany 100: 1322–1331. 10.3732/ajb.1200473. 23748678

[ece371512-bib-0011] Cardinale, B. J. , J. P. Wright , M. W. Cadotte , et al. 2007. “Impacts of Plant Diversity on Biomass Production Increase Through Time Because of Species Complementarity.” Proceedings of the National Academy of Sciences of the United States of America 104: 18123–18128. 10.1073/pnas.0709069104. 17991772 PMC2084307

[ece371512-bib-0012] Carter, B. E. , T. M. Misiewicz , and B. D. Mishler . 2022. “Spatial Phylogenetic Patterns in the North American Moss Flora Are Shaped by History and Climate.” Journal of Biogeography 49: 1327–1338. 10.1111/jbi.14385.

[ece371512-bib-0013] Cassey, P. , T. M. Blackburn , G. J. Russell , K. E. Jones , and J. L. Lockwood . 2004. “Influences on the Transport and Establishment of Exotic Bird Species: An Analysis of the Parrots (Psittaciformes) of the World.” Global Change Biology 10: 417–426. 10.1111/j.1529-8817.2003.00748.x.

[ece371512-bib-0014] Chen, J. , F. Ma , Y. Zhang , C. Wang , and H. Xu . 2021. “Spatial Distribution Patterns of Invasive Alien Species in China.” Global Ecology and Conservation 26: e01432. 10.1016/j.gecco.2020.e01432.

[ece371512-bib-0015] Chen, J. , Y. Zhang , W. Liu , C. Wang , F. Ma , and H. Xu . 2023. “Distribution Patterns and Determinants of Invasive Alien Plants in China.” Plants 12: 2341. 10.3390/plants12122341. 37375966 PMC10300983

[ece371512-bib-0016] Chunco, A. J. 2014. “Hybridization in a Warmer World.” Ecology and Evolution 4: 2019–2031. 10.1002/ece3.1052. 24963394 PMC4063493

[ece371512-bib-0018] Craven, D. , F. Isbell , P. Manning , et al. 2016. “Plant Diversity Effects on Grassland Productivity Are Robust to Both Nutrient Enrichment and Drought.” Philosophical Transactions of the Royal Society, B: Biological Sciences 371: 20150277. 10.1098/rstb.2015.0277. PMC4843698 27114579

[ece371512-bib-0019] Crimmins, T. M. , M. A. Crimmins , and C. D. Bertelsen . 2010. “Complex Responses to Climate Drivers in Onset of Spring Flowering Across a Semi‐Arid Elevation Gradient.” Journal of Ecology 98: 1042–1051. 10.1111/j.1365-2745.2010.01696.x.

[ece371512-bib-0020] Dainese, M. , I. Kühn , and L. Bragazza . 2013. “Alien Plant Species Distribution in the European Alps: Influence of Species' Climatic Requirements.” Biological Invasions 16: 815–831. 10.1007/s10530-013-0540-x.

[ece371512-bib-0021] Domínguez‐Delmás, M. , R. Alejano‐Monge , S. Van Daalen , et al. 2015. “Forest History, Tree‐Rings and Cultural Heritage: Current State and Future Prospects of Dendroarchaeology in the Iberian Peninsula.” Journal of Archaeological Science 57: 180–196. 10.1016/j.jas.2015.02.011.

[ece371512-bib-0022] Dozier, H. 1999. “Plant Introductions and Invasion: History, Public Awareness, and the Case of Ardisiacrenata.” PhD diss., University of Florida.

[ece371512-bib-0023] Duggan, I. C. , C. A. M. Rixon , and H. J. MacIsaac . 2006. “Popularity and Propagule Pressure: Determinants of Introduction and Establishment of Aquarium Fish.” Biological Invasions 8: 377–382. 10.1007/s10530-004-2310-2.

[ece371512-bib-0024] Fanin, N. , M. J. Gundale , M. Farrell , et al. 2018. “Consistent Effects of Biodiversity Loss on Multifunctionality Across Contrasting Ecosystems.” Nature Ecology & Evolution 2: 269–278. 10.1038/s41559-017-0415-0. 29255299

[ece371512-bib-0025] Fick, S. E. , and R. J. Hijmans . 2017. “WorldClim 2: New 1km Spatial Resolution Climate Surfaces for Global Land Areas.” International Journal of Climatology 37: 4302–4315.

[ece371512-bib-0026] Grant, P. R. , and R. Grant . 1992. “Hybridization of Bird Species.” Science 256: 193–197. 10.1126/science.256.5054.193. 17744718

[ece371512-bib-0027] Groves, R. H. 1998. “Recent Incursions of Weeds to Australia 1971–1995.” *CRC for Weed Management Systems Technical Series* 3: 1–74.

[ece371512-bib-0029] Hautier, Y. , F. Isbell , E. T. Borer , et al. 2018. “Local Loss and Spatial Homogenization of Plant Diversity Reduce Ecosystem Multifunctionality.” Nature Ecology & Evolution 2: 50–56. 10.1038/s41559-017-0395-0. 29203922

[ece371512-bib-0031] Higgins, S. I. , and D. M. Richardson . 1999. “Predicting Plant Migration Rates in a Changing World: The Role of Long‐Distance Dispersal.” American Naturalist 153: 464–475. 10.1086/303193. 29578791

[ece371512-bib-0032] Hoffmann, A. A. , and C. M. Sgrò . 2011. “Climate Change and Evolutionary Adaptation.” Nature 470: 479–485. 10.1038/nature09670. 21350480

[ece371512-bib-0033] Hulme, P. E. , S. Bacher , M. Kenis , et al. 2008. “Grasping at the Routes of Biological Invasions: A Framework for Integrating Pathways Into Policy.” Journal of Applied Ecology 45: 403–414. 10.1111/j.1365-2664.2007.01442.x.

[ece371512-bib-0034] Isbell, F. , D. Craven , J. Connolly , et al. 2015. “Biodiversity Increases the Resistance of Ecosystem Productivity to Climate Extremes.” Nature 526: 574–577. 10.1038/nature15374. 26466564

[ece371512-bib-0035] Jacquemyn, H. , R. Brys , O. Honnay , I. Roldán‐Ruiz , B. Lievens , and T. Wiegand . 2012. “Nonrandom Spatial Structuring of Orchids in a Hybrid Zone of Three Orchis Species.” New Phytologist 193: 454–464. 10.1111/j.1469-8137.2011.03913.x. 21955096

[ece371512-bib-0036] Kimura, M. R. , and H. Munehara . 2010. “The Disruption of Habitat Isolation Among Three Hexagrammos Species by Artificial Habitat Alterations That Create Mosaic‐Habitat.” Ecological Research 25: 41–50. 10.1007/s11284-009-0624-3.

[ece371512-bib-0037] Köppen, W. P. 1936. “Das Geographisches System der Klimate.” In Handbuch der Klimatologie, edited by W. P. Köppen and R. Geiger . Gebrueder Borntraeger.

[ece371512-bib-0038] Kreft, H. , and W. Jetz . 2007. “Global Patterns and Determinants of Vascular Plant Diversity.” Proceedings of the National Academy of Sciences of the United States of America 104: 5925–5930. 10.1073/pnas.0608361104. 17379667 PMC1851593

[ece371512-bib-0039] Lamont, B. B. , T. He , N. J. Enright , S. L. Krauss , and B. P. Miller . 2003. “Anthropogenic Disturbance Promotes Hybridization Between *Banksia* Species by Altering Their Biology.” Journal of Evolutionary Biology 16: 551–557. 10.1046/j.1420-9101.2003.00548.x. 14632219

[ece371512-bib-0040] Lavergne, S. , J. Molina , and M. Debussche . 2006. “Fingerprints of Environmental Change on the Rare Mediterranean Flora: A 115‐Year Study.” Global Change Biology 12: 1466–1478. 10.1111/j.1365-2486.2006.01183.x.

[ece371512-bib-0041] Lawrence, E. R. , and D. J. Fraser . 2020. “Latitudinal Biodiversity Gradients at Three Levels: Linking Species Richness, Population Richness and Genetic Diversity.” Global Ecology and Biogeography 29: 770–788. 10.1111/geb.13075.

[ece371512-bib-0042] Liu, J. , M. Li , Q. Zhang , X. Wei , and X. Huang . 2020. “Exploring the Molecular Basis of Heterosis for Plant Breeding.” Journal of Integrative Plant Biology 62: 287–298. 10.1111/jipb.12804. 30916464

[ece371512-bib-0043] Mable, B. K. 2013. “Polyploids and Hybrids in Changing Environments: Winners or Losers in the Struggle for Adaptation?” Heredity 110: 95–96. 10.1038/hdy.2012.105. 23321773 PMC3554446

[ece371512-bib-0044] MacArthur, R. H. , and J. W. MacArthur . 1961. “On Bird Species Diversity.” Ecology 42: 594–598. 10.2307/1932254.

[ece371512-bib-0045] Mack, R. N. 2003. “Global Plant Dispersal, Naturalization, and Invasion: Pathways, Modes, and Circumstances.” In Invasive Species: Vectors and Management Strategies, edited by G. M. Ruiz and J. T. Carlton . Island Press.

[ece371512-bib-0046] Mack, R. N. , and M. Erneberg . 2002. “The United States Naturalized Flora: Largely the Product of Deliberate Introductions.” Annals of the Missouri Botanical Garden 89: 176–189. 10.2307/3298562.

[ece371512-bib-0047] Mallet, J. 2005. “Hybridization as an Invasion of the Genome.” Trends in Ecology & Evolution 20: 229–237. 10.1016/j.tree.2005.02.010. 16701374

[ece371512-bib-0048] Manks, D. D. 1968. “How the American Nursery Trade Began.” In Origins of American Horticulture: A Handbook, edited by D. S. Manks . Brooklyn Botanic Garden.

[ece371512-bib-0050] Marques, D. A. , J. I. Meier , and O. Seehausen . 2019. “A Combinatorial View on Speciation and Adaptive Radiation.” Trends in Ecology & Evolution 34: 531–544. 10.1016/j.tree.2019.02.008. 30885412

[ece371512-bib-0051] Mateo, R. G. , O. Broennimann , S. Normand , et al. 2016. “The Mossy North: An Inverse Latitudinal Diversity Gradient in European Bryophytes.” Scientific Reports 6: 25546. 10.1038/srep25546. 27151094 PMC4858760

[ece371512-bib-0052] Mitchell, N. , L. G. Campbell , J. R. Ahern , K. C. Paine , A. B. Giroldo , and K. D. Whitney . 2019. “Correlates of Hybridization in Plants.” Evolution Letters 3, no. 6: 570–585. 10.1002/evl3.146. 31867119 PMC6906982

[ece371512-bib-0053] Möls, T. , K. Vellak , A. Vellak , and N. Ingerpuu . 2013. “Global Gradients in Moss and Vascular Plant Diversity.” Biodiversity and Conservation 22: 1537–1551. 10.1007/s10531-013-0492-6.

[ece371512-bib-0054] Mooney, H. , and E. Cleland . 2001. “The Evolutionary Impact of Invasive Species.” Proceedings of the National Academy of Sciences of the United States of America 98: 5446–5451. 10.1073/pnas.091093398. 11344292 PMC33232

[ece371512-bib-0055] Moulatlet, G. M. , B. Kusumoto , and J. Pinto‐Ledezma . 2023. “Global Patterns of Phylogenetic Beta‐Diversity Components in Angiosperms.” Journal of Vegetation Science 34: e13203. 10.1111/jvs.13203.

[ece371512-bib-0056] Murata, Y. 2001. “Nationalism, Historical Aspects of: East Asia.” In International Encyclopedia of the Social and Behavioral Sciences, edited by J. S. Neil and B. B. Paul . Pergamon. 10.1016/B0-08-043076-7/02721-2.

[ece371512-bib-0057] Normand, S. , R. E. Ricklefs , F. Skov , J. Bladt , O. Tackenberg , and J. C. Svenning . 2011. “Postglacial Migration Supplements Climate in Determining Plant Species Ranges in Europe.” Proceedings of the Royal Society B: Biological Sciences 278: 3644–3653. 10.1098/rspb.2010.2769. PMC3203492 21543356

[ece371512-bib-0058] Nunn, N. , and N. Qian . 2010. “The Columbian Exchange: A History of Disease, Food, and Ideas.” Journal of Economic Perspectives 24: 163–188. 10.1257/jep.24.2.163.

[ece371512-bib-0059] Parmesan, C. , and G. Yohe . 2003. “A Globally Coherent Fingerprint of Climate Change Impacts Across Natural Systems.” Nature 421: 37–42. 10.1038/nature01286. 12511946

[ece371512-bib-0060] Pfennig, K. S. , A. L. Kelly , and A. A. Pierce . 2016. “Hybridization as a Facilitator of Species Range Expansion.” Proceedings of the Royal Society B: Biological Sciences 283: 20161329. 10.1098/rspb.2016.1329. PMC5046898 27683368

[ece371512-bib-0061] Pyšek, P. , J. Sadlo , and B. Mandak . 2002. “Catalogue of Alien Plants of the Czech Republic.” Preslia 74: 97–186.

[ece371512-bib-0062] Qian, H. , T. Deng , Y. Jin , L. Mao , D. Zhao , and R. E. Ricklefs . 2019. “Phylogenetic Dispersion and Diversity in Regional Assemblages of Seed Plants in China.” Proceedings of the National Academy of Sciences of the United States of America 116: 23192–23201. 10.1073/pnas.1822153116. 31659037 PMC6859352

[ece371512-bib-0063] Qian, H. , and S. Qian . 2023. “Geographic Patterns of Taxonomic and Phylogenetic β‐Diversity of Angiosperm Genera in Regional Floras Across the World.” Plant Diversity 45: 491–500. 10.1016/j.pld.2023.07.008. 37936816 PMC10625901

[ece371512-bib-0064] Reich, P. B. , D. Tilman , F. Isbell , et al. 2012. “Impacts of Biodiversity Loss Escalate Through Time as Redundancy Fades.” Science 336: 589–592. 10.1126/science.1217909. 22556253

[ece371512-bib-0065] Ren, H. , K. A. Yurkonis , L. Wang , et al. 2022. “Temporal Stabilizing Effects of Species Richness and Seed Arrangement on Grassland Biomass Production.” Journal of Ecology 110: 1606–1614. 10.1111/1365-2745.13895.

[ece371512-bib-0066] Rhymer, J. M. , and D. Simberloff . 1996. “Extinction by Hybridization and Introgression.” Annual Review of Ecology, Evolution, and Systematics 27: 83–109. 10.1146/annurev.ecolsys.27.1.83.

[ece371512-bib-0067] Rieseberg, L. H. , S. J. Baird , and K. A. Gardner . 2000. “Hybridization, Introgression, and Linkage Evolution.” Plant Molecular Biology 42: 205–224. 10.1023/A:1006340407546. 10688138

[ece371512-bib-0068] Root, T. L. , D. P. MacMynowski , M. D. Mastrandrea , and S. H. Schneider . 2005. “Human‐Modified Temperatures Induce Species Changes: Joint Attribution.” Proceedings of the National Academy of Sciences of the United States of America 102: 7465–7469. 10.1073/pnas.0502286102. 15899975 PMC1129055

[ece371512-bib-0069] Royle, S. A. 2009. “Exploration.” In International Encyclopedia of Human Geography, edited by R. Kitchin and N. Thrift . Elsevier. 10.1016/B978-008044910-4.00357-6.

[ece371512-bib-0070] Schnabel, F. , X. Liu , M. Kunz , et al. 2021. “Species Richness Stabilizes Productivity via Asynchrony and Drought‐Tolerance Diversity in a Large‐Scale Tree Biodiversity Experiment.” Science Advances 7: eabk1643. 10.1126/sciadv.abk1643. 34919425 PMC8682986

[ece371512-bib-0071] Schuldt, A. , T. Assmann , M. Brezzi , et al. 2018. “Biodiversity Across Trophic Levels Drives Multifunctionality in Highly Diverse Forests.” Nature Communications 9: 2989. 10.1038/s41467-018-05421-z. PMC6068104 30065285

[ece371512-bib-0072] Seehausen, O. 2004. “Hybridization and Adaptive Radiation.” Trends in Ecology & Evolution 19: 198–207. 10.1016/j.tree.2004.01.003. 16701254

[ece371512-bib-0073] Semmens, B. X. , E. R. Buhle , A. K. Salomon , and C. V. Pattengill‐Semmens . 2004. “A Hotspot of Non‐Native Marine Fishes: Evidence for the Aquarium Trade as an Invasion Pathway.” Marine Ecology Progress Series 266: 239–244. 10.3354/meps266239.

[ece371512-bib-0074] Soltis, P. S. , and D. E. Soltis . 2009. “The Role of Hybridization in Plant Speciation.” Annual Review of Plant Biology 60: 561–588. 10.1146/annurev.arplant.043008.092039. 19575590

[ece371512-bib-0075] Staude, I. R. , and J. Ebersbach . 2023. “Neophytes May Promote Hybridization and Adaptations to a Changing Planet.” Ecology and Evolution 13: e10405. 10.1002/ece3.10405. 37593753 PMC10427993

[ece371512-bib-0076] Sujii, P. S. , S. Cozzolino , and F. Pinheiro . 2019. “Hybridization and Geographic Distribution Shapes the Spatial Genetic Structure of Two Co‐Occurring Orchid Species.” Heredity 123: 458–469. 10.1038/s41437-019-0254-7. 31391556 PMC6781141

[ece371512-bib-0077] Taylor, S. A. , E. L. Larson , and R. G. Harrison . 2015. “Hybrid Zones: Windows on Climate Change.” Trends in Ecology & Evolution 30: 198–406. 10.1016/j.tree.2015.04.010. PMC4794265 25982153

[ece371512-bib-0078] Thakur, M. P. , W. H. Putten , R. A. Wilschut , et al. 2021. “Plant‐Soil Feedbacks and Temporal Dynamics of Plant Diversity‐Productivity Relationships.” Trends in Ecology & Evolution 36: 651–661. 10.1016/j.tree.2021.03.011. 33888322

[ece371512-bib-0079] Thomas, C. D. 2015. “Rapid Acceleration of Plant Speciation During the Anthropocene.” Trends in Ecology & Evolution 30: 448–455. 10.1016/j.tree.2015.05.009. 26115931

[ece371512-bib-0080] Tilman, D. , P. B. Reich , and J. M. Knops . 2006. “Biodiversity and Ecosystem Stability in a Decade‐Long Grassland Experiment.” Nature 441: 629–632. 10.1038/nature04742. 16738658

[ece371512-bib-0081] Tutin, T. G. , V. H. Heywood , N. A. Burges , et al. 1980. Flora Europaea. 5 volumes. Cambridge University Press.

[ece371512-bib-0082] Vallejo‐Marín, M. , and S. J. Hiscock . 2016. “Hybridization and Hybrid Speciation Under Global Change.” New Phytologist 211: 1170–1187. 10.1111/nph.14004. 27214560

[ece371512-bib-0083] Van Moorsel, S. J. , T. Hahl , O. L. Petchey , et al. 2021. “Co‐Occurrence History Increases Ecosystem Stability and Resilience in Experimental Plant Communities.” Ecology 102: e03205. 10.1002/ecy.3205. 32979225

[ece371512-bib-0084] Viljoen, A. M. , B. Demirci , K. H. C. Baser , et al. 2006. “Microdistillation and Essential Oil Chemistry – A Useful Tool for Detecting Hybridisation in Plectranthus (Lamiaceae).” South African Journal of Botany 72: 99–104. 10.1016/j.sajb.2005.05.003.

[ece371512-bib-0085] Wagg, C. , M. J. O'Brien , A. Vogel , et al. 2017. “Plant Diversity Maintains Long‐Term Ecosystem Productivity Under Frequent Drought by Increasing Short‐Term Variation.” Ecology 98: 2952–2961. 10.1002/ecy.2003. 28869781

[ece371512-bib-0086] Wagg, C. , C. Roscher , A. Weigelt , et al. 2022. “Biodiversity–Stability Relationships Strengthen Over Time in a Long‐Term Grassland Experiment.” Nature Communications 13: 7752. 10.1038/s41467-022-35189-2. PMC9751076 36517483

[ece371512-bib-0087] Walters, D. M. , M. J. Blum , B. Rashleigh , B. J. Freeman , B. A. Porter , and N. M. Burkhead . 2008. “Red Shiner Invasion and Hybridization With Blacktail Shiner in the Upper Coosa River, USA.” Biological Invasions 10: 1229–1242. 10.1007/s10530-007-9198-6.

[ece371512-bib-0088] Walther, G. R. , S. Berger , and M. T. Sykes . 2005. “An Ecological ‘Footprint’ of Climate Change.” Proceedings of the Royal Society B: Biological Sciences 272: 1427–1432. 10.1098/rspb.2005.3119. PMC1559830 16011916

[ece371512-bib-0089] Wang, X. , Y. Ge , S. Gao , et al. 2021. “Evenness Alters the Positive Effect of Species Richness on Community Drought Resistance via Changing Complementarity.” Ecological Indicators 133: 108464. 10.1016/j.ecolind.2021.108464.

[ece371512-bib-0090] Whitney, K. D. , J. R. Ahern , L. G. Campbell , L. P. Albert , and M. S. King . 2010. “Patterns of Hybridization in Plants.” Perspectives in Plant Ecology, Evolution and Systematics 12: 175–182. 10.1016/j.ppees.2010.02.002.

[ece371512-bib-0091] Wiegand, K. M. 1935. “A Taxonomist's Experience With Hybrids in the Wild.” Science 81: 161–166. 10.1126/science.81.2094.161. 17817007

[ece371512-bib-0092] Yakimowski, S. B. , and L. H. Rieseberg . 2014. “The Role of Homoploid Hybridization in Evolution: A Century of Studies Synthesizing Genetics and Ecology.” American Journal of Botany 101: 1247–1258. 10.3732/ajb.1400201. 25156978

[ece371512-bib-0093] Yang, G. , C. Wagg , S. D. Veresoglou , S. Hempel , and M. C. Rillig . 2018. “How Soil Biota Drive Ecosystem Stability.” Trends in Plant Science 23: 1057–1067. 10.1016/j.tplants.2018.09.007. 30287162

[ece371512-bib-0094] Zhang, Y. , L. Qian , X. Chen , et al. 2022. “Diversity Patterns of Cushion Plants on the Qinghai‐Tibet Plateau: A Basic Study for Future Conservation Efforts on Alpine Ecosystems.” Plant Diversity 44: 231–242. 10.1016/j.pld.2021.09.001. 35769589 PMC9209862

